# Supplementary material for: The interactome of intact mitochondria by cross-linking mass spectrometry provides evidence for coexisting respiratory supercomplexes
Source: Mol Cell Proteomics. 2017 Dec 8;17(2):216–32. doi: 10.1074/mcp.RA117.000470 (PMC5795388; doi:10.1074/mcp.RA117.000470)
Supplement: Supplemental Data [file supp_RA117.000470_133922_0_supp_23978_fzffwf.zip › spectra_annotation/mito_DR_spectra_annotation/468-1-1-1-4-1.pdf]

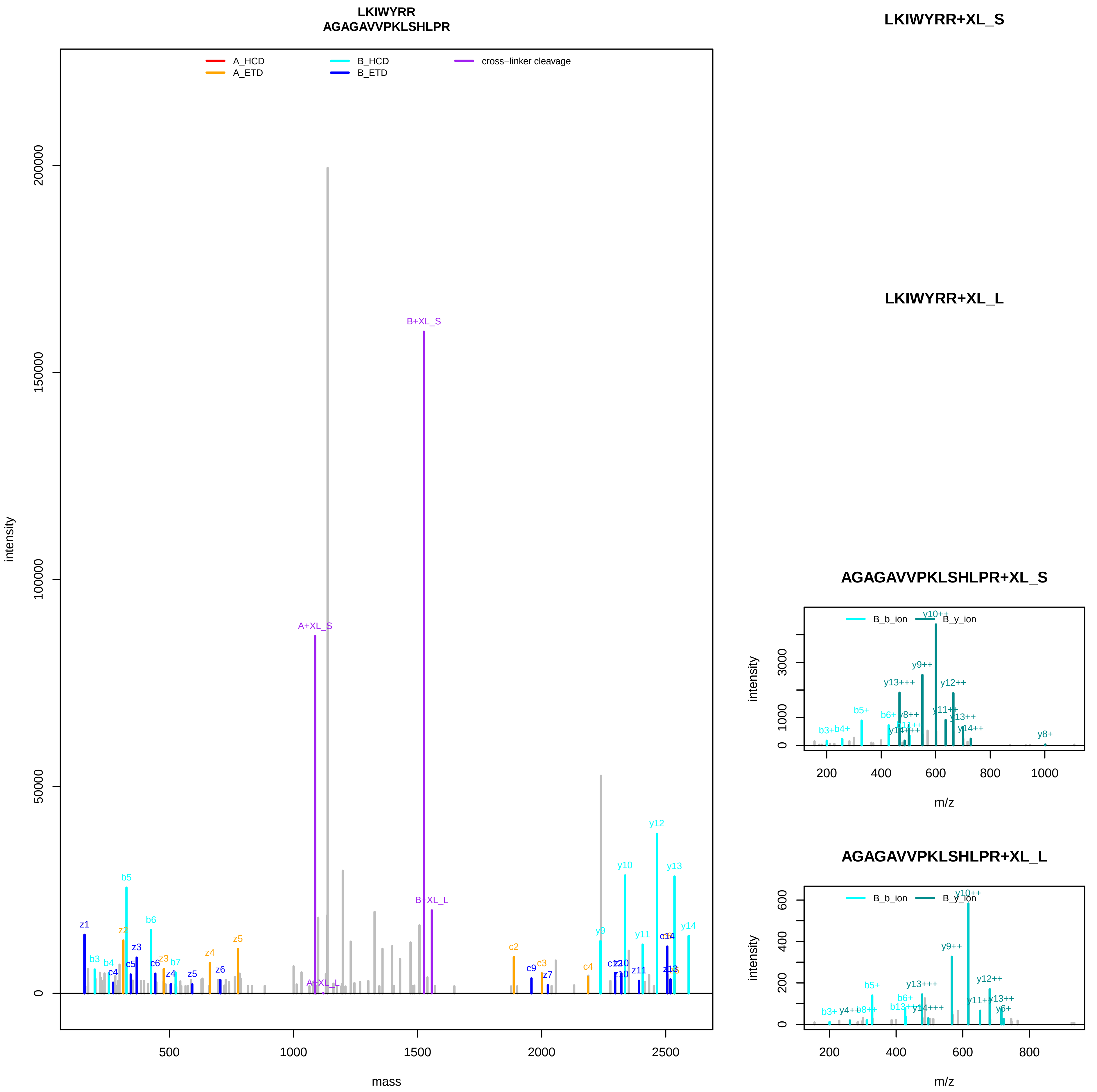
LKIWYRR
AGAGAVVPKLSHLPR
A_HCD
A_ETD
B_HCD
B_ETD
cross−linker cleavage
intensity
mass
0
50000
100000
150000
200000
500
1000
1500
2000
2500
B+XL_S
A+XL_S
B+XL_L
A+XL_L
z1
b3
b4
c4
b5
z2
z3
c5
b6
c6
z3
b7
z4
z5
z4
z6
z5
c2
c9
c3
z7
c4
y9
y10
y11
y12
y13
y14
z11
c14
z13
LKIWYRR+XL_S
LKIWYRR+XL_L
AGAGAVVPKLSHLPR+XL_S
B_b_ion
B_y_ion
intensity
m/z
0
1000
3000
200
400
600
800
1000
y10++
y9++
y13+++
y12++
b5+
b6+
y8++
y11++
y13++
y14++
y8+
b3+
b4+
AGAGAVVPKLSHLPR+XL_L
B_b_ion
B_y_ion
intensity
m/z
0
200
400
600
800
y10++
y9++
b5+
y13+++
y12++
b6+
b13+++
y14+++
y11++
y13++
y6+
b3+
y4++
b8++
